# Timely Supplementation of Hydrogels Containing Sulfated or Unsulfated Chondroitin and Hyaluronic Acid Affects Mesenchymal Stromal Cells Commitment Toward Chondrogenic Differentiation

**DOI:** 10.3389/fcell.2021.641529

**Published:** 2021-04-12

**Authors:** Nicola Alessio, Antonietta Stellavato, Domenico Aprile, Donatella Cimini, Valentina Vassallo, Giovanni Di Bernardo, Umberto Galderisi, Chiara Schiraldi

**Affiliations:** ^1^Department of Experimental Medicine, Luigi Vanvitelli Campania University, Naples, Italy; ^2^Sbarro Institute for Cancer Research and Molecular Medicine, Center for Biotechnology, Temple University, Philadelphia, PA, United States; ^3^Genome and Stem Cell Center (GENKOK), Erciyes University, Kayseri, Turkey

**Keywords:** extractive sulfated chondroitin, bio-fermentative unsulphated chondroitin, hyaluronic acid, mesenchymal stromal cells, differentiation chondrocytes regeneration

## Abstract

Mesenchymal stromal cells (MSCs) are currently used for cartilage cell therapy because of their well proven capacity to differentiate in chondrocytes. The advantage of MSC-based therapy is the possibility of producing a high number of chondrocytes for implants. The transplant procedure, however, has some limitations, since MSCs may produce non-functional chondrocytes. This limit has been challenged by cultivating MSC in media with hydrogels containing hyaluronic acid (HA), extractive chondroitin sulfate (CS), or bio-fermentative unsulphated chondroitin (BC) alone or in combination. Nevertheless, a clear study of the effect of glycosaminoglycans (GAGs) on chondrocyte differentiation is still lacking, especially for the newly obtained unsulfated chondroitin of biotechnological origin. Are these GAGs playing a role in the commitment of stem cells to chondrocyte progenitors and in the differentiation of progenitors to mature chondrocytes? Alternatively, do they have a role only in one of these biological processes? We evaluated the role of HA, CS, and – above all – BC in cell commitment and chondrocyte differentiation of MSCs by supplementing these GAGs in different phases of *in vitro* cultivation. Our data provided evidence that a combination of HA and CS or of HA and BC supplemented during the terminal *in vitro* differentiation and not during cell commitment of MSCs improved chondrocytes differentiation without the presence of fibrosis (reduced expression of Type I collagen). This result suggests that a careful evaluation of extracellular cues for chondrocyte differentiation is fundamental to obtaining a proper maturation process.

## Introduction

Our joints are endowed with articular cartilage to tolerate daily physical and mechanical stress. The articular cartilage contains chondrocytes that are surrounded by collagens, different types of glycosaminoglycans (GAGs) and proteoglycans (Davies and Kuiper, 2019). The homeostasis and tissue repair of cartilage is inefficient due to the lack of blood vessels and the sparse, isolated chondrocytes. This condition renders cartilage prone to degeneration due to the onset of diseases, such as osteoarthritis (OA), a disabling and painful pathology, which affects a great number of individuals in their 50s and beyond (Dulay et al., 2015). This disease results from progressive degradation of cartilage with loss of Type II collagen and of GAGs, such as hyaluronic acid (HA) and chondroitin sulfate (CS) (Dulay et al., 2015; Fonsi et al., 2020). Several therapeutic options exist for treating OA. Some of them may give durable results, while others may provide temporary relief from OA symptoms and pain (Dulay et al., 2015; Davies and Kuiper, 2019; Fonsi et al., 2020). Indeed, there is no gold standard treatment for OA. It is important for physicians to evaluate the general health conditions and individual needs of every patient (hobbies, lifestyle). The intra-articular injection of mixtures containing either HA or CS or HA + CS have already been used or are in clinical trial evaluation (Pederzini et al., 2013; Kloppenburg, 2014; Rivera et al., 2016).

At a certain stage of OA, physicians agree with some international guidelines released by the European Society for Clinical and Economic Aspects of Osteoporosis and Osteoarthritis (ESCEO) or the European League Against Rheumatism (EULAR) that visco-supplementation may play a positive role, especially in improving joint movement. This is mainly obtained through the injection of HA-based gels, which are for the most part consistent in linear HA. More recently complexes made by high and low molecular weight (MW) HA have been used permitting a higher amount delivered per injection, while few products are based on crosslinked or chemical-stabilized HA. This relatively poor invasive medical approach may give therapeutic effects that can last some months (Fonsi et al., 2020). In recent decades, surgical approaches aiming at repairing cartilage have been implemented. Among these options, the autologous chondrocyte implantation (ACI) procedure has been used since the early years of this century. The ACI is a re-implant of *in vitro* expanded autologous chondrocytes into the damaged joint. The first ACI treatments suffered from technical challenges associated with avoiding tearing of the periosteum. This problem has been solved by replacing periosteum with porcine collagen membranes or even better with the implant of chondrocytes embedded into hydrated scaffolds containing GAGs (Choi et al., 2010; Davies and Kuiper, 2019). An ACI transplant with scaffold implantation for chondral defects appears to be a safe and effective procedure for both decreasing pain and improving cartilage function. Nevertheless, this approach has some limitations. The freshly extracted chondrocytes obtainable from patients are not sufficient for re-implants and hence these cells have to be cultivated *in vitro* to increase their number. Anyway, the *in vitro* proliferation potential of chondrocytes is low, and chondrocytes may dedifferentiate during cultivation or even acquire unwanted phenotypic features (e.g., production of Type I collagen) (Choi et al., 2010; Davies and Kuiper, 2019).

Several scientific and clinical studies have shown that mesenchymal stromal cells (MSCs), obtained from patients’ bone marrow or adipose tissue, may represent a valid alternative to ACI (Wang et al., 2019). MSCs are a heterogeneous population present in the stromal component of many tissues and organs, mainly in bone marrow and adipose tissue (Galderisi and Giordano, 2014). MSCs contain stem cells that are able to differentiate into osteocytes, adipocytes and chondrocytes and also secrete a lot of cytokines, growth factors and immunomodulators. For these reasons, MSCs play a key role in organismal homeostasis and tissue repair (Galderisi and Giordano, 2014). MSCs have been cultivated *in vitro* and used for cartilage cell therapy because of their well proven capacity to differentiate among chondrocytes. The advantage of MSC-based therapy is the possibility of producing a high number of chondrocytes for implants. An alternative use of MSCs for OA treatment is the implant of undifferentiated MSCs within damaged cartilage areas. This approach relies on the immunomodulatory and pro-growth properties of factors that are released by MSCs that can promote cartilage repair by stimulation of endogenous cells (Wang et al., 2019). MSC transplants do have some limitations. For example, MSCs may produce non-functional chondrocytes with the production of extracellular matrix (ECM) components that are typical of a fibrotic and hypertrophic cartilage (Wang et al., 2019). Several research studies have tried to overcome such a limit by improving MSC cultivation and implants by using hydrogels containing GAGs, collagens and other polymers (polylactic acids, polyglycolic acids, gelatin, etc.). Either MSCs or chondrocytes have been cultivated and/or implanted with hydrogels of chondroitin or HA alone or in combinations. Nevertheless, a clear study on the effect of GAGs on chondrocytes differentiation is still lacking. Does any GAG play a role in the commitment of stem cells to chondrocyte progenitors and in the differentiation of progenitors to mature chondrocytes? Alternatively, does any of the GAGs’ affect (at least) one of the above-described biological processes? These questions are fundamental for a proper and “smart” application of hydrogels in the procedures for cartilage repair. Furthermore, of no less importance, treatments with hydrogels should reduce the onset of senescence to safeguard the therapeutic potential of any MSC batch destined for patients, as we described in a previous investigation (Alessio et al., 2018b).

In this context, we decided to investigate the effects of HA and the diverse sulfated and unsulfated chondroitins on the *in vitro* commitment and differentiation of MSCs in chondrocytes to evaluate the contribution of these GAGs to these processes.

## Results

**FIGURE 1:**
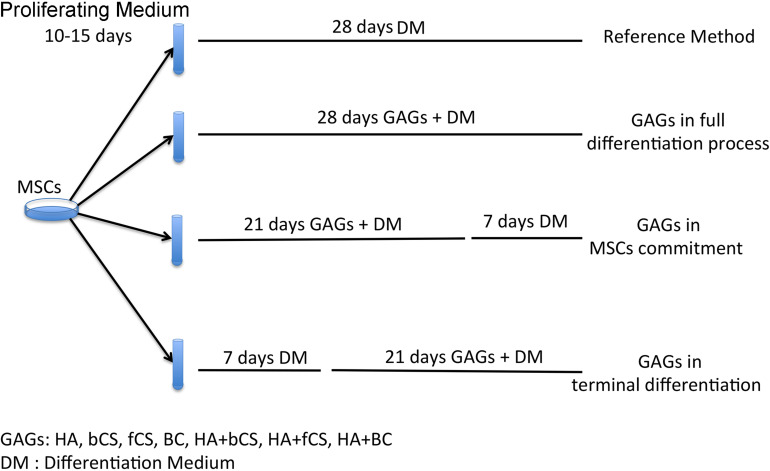
Experimental plan. MSCs were expanded for 10–15 days in a PM. Then we induced chondrocyte differentiation with four different protocols. The reference method was a procedure with a differentiation medium (DM) containing differentiating factors. The other three methods were based on DM supplemented with GAGs at different time points during the differentiation process.

The stemness (stem cell properties), cell commitment and differentiation of MSCs are regulated by ECM and physical and chemical cues. Changes in ECM composition may affect MSC biological functions by either promoting lineage selection and/or differentiation or maintaining undifferentiated status (Saidova and Vorobjev, 2020). In this context, we decided to evaluate the role of HA, CS and beyond BC in cell commitment and chondrocyte differentiation of MSCs by using the experimental approach depicted in Figure 1. In brief, MSCs were cultivated in a proliferating medium (PM) and then this was exchanged for a differentiating medium (DM) containing signaling molecules known to induce chondrocyte lineage selection and differentiation (Solchaga et al., 2011). The *in vitro* commitment and differentiation with DM lasted 28 days, and this procedure was considered our reference method. In an alternative experimental design, DM was supplemented with hydrogels containing either HA, CS, or BC, or a combination of HA with the chondroitins. Hydrogels (0.16% final concentration) were added at the beginning of *in vitro* differentiation and their supplementation to DM continued every three days till the 28th day of cultivation. We named this method *GAGs in full differentiation process*. Alternatively, we supplemented DM with hydrogels for 21 days and then continued incubation with DM only for a further 7 days. This method was termed *GAGs in MSC commitment*. We also grew cells in DM for 7 days and then added hydrogels to DM and continued incubation for a further 21 days. The rationale of our experimental protocol takes into consideration that the absence of proliferation is a pre-requisite for terminal differentiation of MSCs in chondrocytes. According to several findings, the terminal differentiation of MSCs may start around the 21th day of *in vitro* differentiation when no signs of cell proliferation are detected (Ichinose et al., 2010; Dexheimer et al., 2012). We selected the 21th day as the beginning of terminal differentiation and hence evaluated the effect of GAG supplementation during the cell commitment (21 days) or later during terminal differentiation. This method was named *GAGs in MSC terminal differentiation*.

Chondroitin sulfate is a glycosaminoglycan composed of repeating disaccharide units of D-glucuronic acid and N-acetyl-D-galactosamine and has different sulfation profiles depending mainly on the extractive source. The most common commercially available CS derives either from marine organisms (sharks and skates) or from pigs, bovine or chicken. The marine-derived CS has a higher MW (30–80 kDa) and a different sulfation compared with CS extracted from terrestrial animals, that also present a lower size (14–26 kDa in MW) (Restaino et al., 2019). A new source of CS are genetically modified bacteria that can produce high purity unsulfated biotechnological chondroitin (Schiraldi et al., 2012). The sulfation pattern of CS can promote different biological effects (Nandini and Sugahara, 2006). We then decided to evaluate the impact of three different CSs on *in vitro* cell commitment and chondrocyte differentiation of MSCs. We used CS obtained from bovine (bCS) or (fCS) fish and compared these to bio-fermentative, unsulfated chondroitin (BC) obtained from engineered bacteria and extensively purified to obtain a pharma grade biomolecule suitable for preclinical studies (Stellavato et al., 2016).

The *in vitro* commitment and differentiation of MSCs in chondrocyte can be evaluated by determining the expression values of a few markers by quantitative RT-PCR (Herlofsen et al., 2011). Some of these markers present an expression kinetic showing a progressive increase in gene expression during *in vitro* commitment and differentiation, while others have more complex and non-uniform expression profiles (Herlofsen et al., 2011). Then, we evaluated MSC maturation in 22 different experimental conditions by assessing the expression levels of aggrecan, Type II collagen and SOX9, which show a progressive accumulation during *in vitro* commitment and differentiation. We also measured the Type I collagen expression, since it has been demonstrated that this collagen’s isoform is associated with OA onset and related fibrosis (Miosge et al., 2004). Indeed, a limit of many *in vitro* chondrocyte differentiation protocols is due to production of mature cells with altered phenotype showing increased levels of Type I collagen (Miosge et al., 2004; Lee and Wang, 2017).

In this context, our experimental algorithm aimed to assess if and to what extent our cell commitment and differentiation protocols based on supplementation of DM with GAGs produced an increase in aggrecan, Type II collagen and SOX9 compared with the reference differentiation method. Preliminary, we evaluated the effectiveness of reference differentiation procedure in our experimental condition (Supplementary file 1). We also ascertained whether a switch from Type II to Type I collagen occurred. This analysis was carried out at the end of the 4-week *in vitro* differentiation. The supplementation of GAGs for the entire cell commitment and differentiation process (28 days) did not evidence significant differences in the selected markers compared with the reference method (data not shown).

**FIGURE 2:**
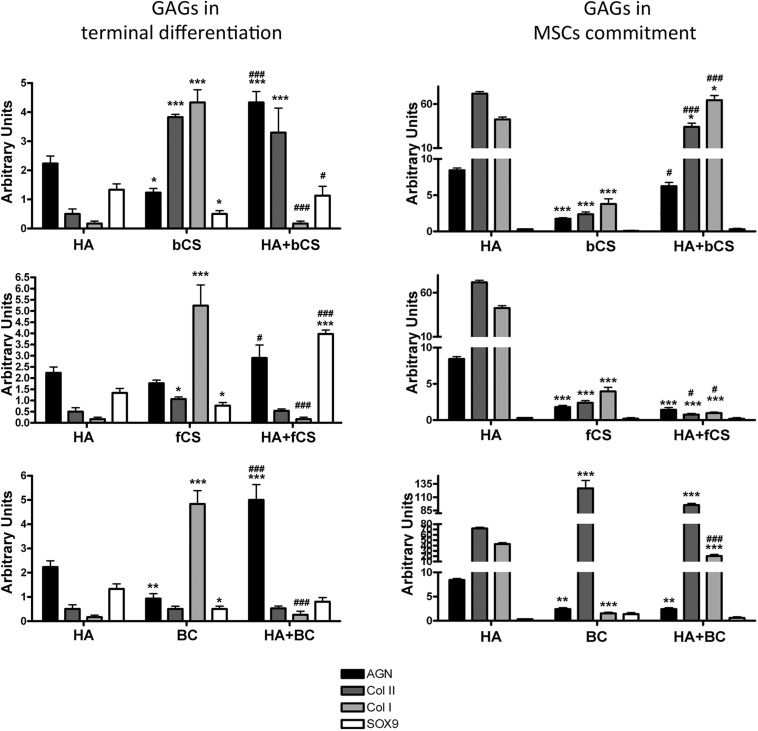
Chondrocyte differentiation markers in several experimental conditions. The picture shows the mRNA levels of aggrecan (AGN), Type I collagen (Col I), Type II collagen (Col II), and SOX9 in MSCs induced to chondrocyte differentiation with GAGs in terminal differentiation and GAGs in MSC commitment, respectively. Data are normalized to mRNA levels observed in differentiated cultures obtained with the Reference Method. For each mRNA, the expression level observed in reference cultures is set at 1, its decrease or increase in the other experimental conditions is expressed as fold change. Data are reported with standard deviation (*n* = 3). We compared the gene expression in HA hydrogel with the HA + bCS hydrogel. In each histogram the symbol * indicates the statistical difference between them. We compared the gene expression in bCS hydrogel with the HA + bCS hydrogel. In each histogram the symbol ^#^ indicates the statistical difference between them (*< 0.05; **< 0.01; ***< 0.001; ^#^< 0.05; ^##^< 0.01; ^###^< 0.001).

We then evaluated the effect of GAG supplementation during the first phases of *in vitro* commitment and differentiation (GAGs in MSC commitment) or in the final maturation stage (GAGs in MSC terminal differentiation) (Figure 2). The GAGs in MSC terminal differentiation protocol showed significant changes compared with the reference method (Figure 2). The HHA promoted an increase of aggrecan and Type II collagen. The two different CSs and BC also promoted upregulation of these two markers, but their supplementation induced a strong upregulation of Type I collagen (Figure 2). Of note, the combination of HHA with either bCS or fCS or BC induced strong upregulation of differentiation markers and no modification in Type I collagen expression (Figure 2). This outcome is associated with no modification of the fibrosis marker, the Type I collagen.

The GAGs in MSC commitment protocol produced different results (Figure 2). The HHA alone produced a huge upregulation of Type II collagen (40 times). This was associate with an increase in aggrecan and a strong upregulation of Type I collagen (eight times). Among the two CSs and BC, only this latter one induced the highest increase in Type II collagen, but no changes were detected in the other differentiation markers. The combination of HHA with one of two different CSs or with BC did not give impressive changes in the expression profiles of the analyzed markers (Figure 2).

The described results may suggest that a “specific temporal window” is fundamental for GAG supplementation during *in vitro* commitment and chondrocyte differentiation of MSCs. In detail, the GAGs seem to have a role in MSC terminal differentiation and not for the entire differentiation period or for commitment. This hypothesis is strengthened by a supplemental experiment performed. MSCs were cultivated in proliferating conditions with HHA or CSs either alone or in combination. This investigation aimed to ascertain if the GAGs can promote cell commitment or differentiation in absence of other chemical cues, such as those present in DM. The RT-PCR data showed that this was not the case: in all the different GAG combinations we observed a significant increase of the fibrotic marker Type I collagen (data not shown).

The GAGs in MSC terminal differentiation protocol gave the best results for chondrocyte differentiation markers; hence, we decided to further confirm this outcome by RT-PCR analysis of HAS-1 expression, immunohistochemistry (IHC) detection of Type I collagen, Type II collagen, and MMP13 along with apoptosis assay.

**FIGURE 3:**
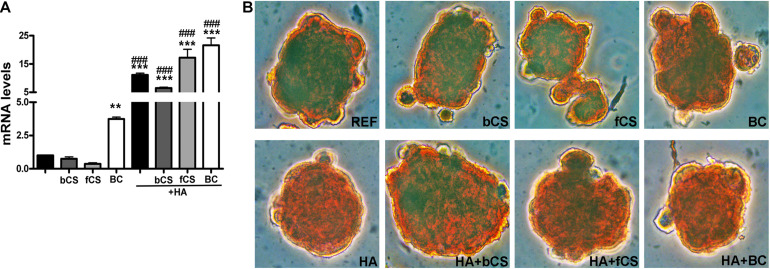
HAS-1 expression and Safranin O staining. **(A)** The histogram shows the HAS-1 mRNA levels in MSCs induced to chondrocyte differentiation with GAGs in terminal differentiation. Data are normalized to mRNA levels observed in differentiated cultures obtained with the Reference Method. For each mRNA, the expression level observed in reference cultures is set at 1, its decrease or increase in the other experimental conditions is expressed as fold change. Data are reported with standard deviation (*n* = 3) (–) stands for no chondroitin supplementation. We compared the gene expression in HA hydrogel with the HA + bCS hydrogel. In each histogram the symbol * indicates the statistical difference between them. We compared the gene expression in bCS hydrogel with the HA + bCS hydrogel. In each histogram the symbol ^#^ indicates the statistical difference between them (* < 0.05; ** < 0.01; *** < 0.001; ^#^ < 0.05; ^##^ < 0.01; ^###^ < 0.001). **(B)** Representative images of Safranin O staining on whole-pellet culture samples. In the first row, REF indicates the reference differentiation. All the other experimental conditions were with the procedure we named “GAGs in terminal differentiation.” In the first row, the bCS, fCS, and BC indicate differentiation with three different chondroitin hydrogels. In the second row, the GAG terminal differentiation was performed with HA alone (leftmost) or in combination with the chondroitin molecules.

HA is synthesized by an enzyme called hyaluronan synthase (HAS) and its expression is lower in *in vitro* differentiated chondrocytes compared to explanted chondrocytes if the differentiation procedure is suboptimal (Ono et al., 2013). We evaluated the mRNA level of HAS-1 isoform and found that the hydrogel with HA and BC showed the highest expression of *HAS-1* gene compared to reference differentiation (Figure 3A).

**FIGURE 4:**
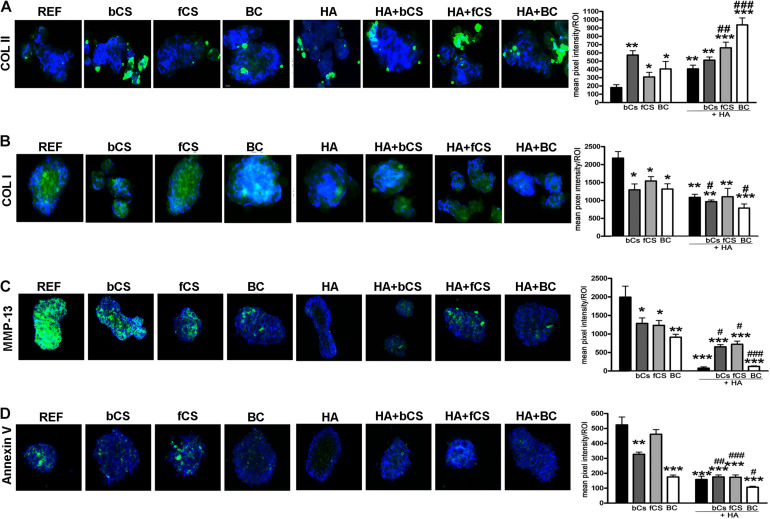
Immunohistochemistry on differentiated MSCs. **(A–C)** Representative images of Type II collagen **(A)**, Type I collagen **(B)**, and MMP13 **(C)** immunostaining (green) on pellets treated as designated in the figure. Nuclei were counterstained with DAPI (blue). Graphs show the mean pixel intensity of green staining. The staining was quantified using Quantity One 1-D analysis software (Bio-Rad, CA, United States). Data are expressed as ROI (region of interest) units with standard deviation (*n* = 3, *< 0.05; **< 0.01; ***< 0.001; ^#^< 0.05; ^##^< 0.01; ^###^< 0.001). The symbol * indicates the significance between reference differentiation and all the other experimental conditions. The symbol ^#^ indicates significance between each hydrogel with chondroitin only and the corresponding hydrogel with chondroitin plus HA (–) stands for no chondroitin supplementation. **(D)** Representative image of apoptosis detection by annexing staining (green) on pellets treated as designated in the figure. Nuclei were counterstained with DAPI (blue). Graphs show the mean pixel intensity of green staining. Data are expressed with standard deviation (*n* = 3, *< 0.05; **< 0.01; ***< 0.001; ^#^< 0.05; ^##^< 0.01; ^###^< 0.001). The symbol * indicates the significance between reference differentiation and all the other experimental conditions. The symbol ^#^ indicates significance between each hydrogel with chondroitin only and the corresponding hydrogel with chondroitin plus HA. NG stands for No GAG supplementation.

We performed an IHC study to strengthen data obtained with mRNA analysis. IHC was carried out on whole-cell pellets, since a reduced number of cells was used for differentiation procedures, given the high number of different experimental conditions analyzed in the present study. The Safranin staining showed that HA alone or in combination with the different chondroitin moieties promoted chondrocyte differentiation with accumulation of GAGs and Type II collagen, as evidenced by red staining (Figure 3B). Further insights were obtained with the immunostaining for Type II and Type I collagen (Figures 4A,B). The HA/BC hydrogel promoted the highest production of Type II collagen and the lowest expression of Type I collagen (Figures 4A,B). The absence of deregulated differentiation was evaluated with MMP13, since its expression is found associated with chondrocyte hypertrophy (D’Angelo et al., 2000). The reference differentiation method produced chondrocytes with the highest MMP13 expression, and this is in line with high Type I collagen expression (Figure 4C). The HA alone or in combination with the two CSs or the BC induced low MMP13 expression (Figure 4C). The *in vitro* expansion and/or differentiation of cells for transplants should be optimized to produce viable and functional cells. In this context, low levels of cell death are highly desirable. We evaluated apoptosis in the several differentiation procedures and found that HA/BC hydrogel gave the lowest apoptosis rate (Figure 4D).

## Discussion

Natural and synthetic scaffolds are widely used in cartilage cell therapy. The HA is a very popular scaffold since it can promote chondrocyte growth and ECM production. For example, HYAFF-11 (Fidia Advanced Biopolymers, Italy) is an HA-based scaffold that has proved effective for ACI. Besides its effects on mature chondrocytes, HA can support the proliferation of MSCs and their differentiation in chondrocyte and can delay onset of replicative senescence during *in vitro* cultivation (Alessio et al., 2018b; Davies and Kuiper, 2019). CS from different sources is another promising GAG for cartilage cell therapy (Hui et al., 2007; Bedini et al., 2011; Stellavato et al., 2016; Davies and Kuiper, 2019). Nevertheless, the use of GAGs for *in vitro* MSC differentiation to obtain mature chondrocytes or for *in vitro* expansion of explanted chondrocytes showed some challenges. MSCs can differentiate in chondrocytes with fibrotic features or chondrocytes tend to dedifferentiate during cultivation. These issues have not been definitively addressed by the numerous findings in the field and contrasting results have been published on the effects of GAGs for chondrocyte differentiation. The discrepancies have been attributed to differences in cultivation protocols and/or in concentration and purity of GAGs in cultures. We used a different approach: we considered the fact that the extracellular environment where stem cells reside is not static but changes continuously to address endogenous and exogenous stimuli. In this context, a GAG component of ECM may be important for cell commitment and dispensable for other cell phases or even its presence may block the passage from a cell status to a new one.

We evaluated the role of different chondroitin molecules and HA in cell commitment and chondrocyte differentiation of MSCs by supplementing these GAGs in different phases of *in vitro* cultivation. In our experimental plan we also evaluated the effect of three different CSs that present diversity in their MW and sulfation pattern.

Our data demonstrated that a combination of HA and chondroitin supplemented during the terminal *in vitro* differentiation and not during cell commitment of MSCs produce the best result in terms of chondrocyte differentiation without the presence of fibrosis (reduced expression of Type I collagen). This result suggests that a careful evaluation of extracellular cues for chondrocyte differentiation is fundamental to obtaining a proper maturation process.

We noticed that the combination of HA with chondroitin gave better results than supplementation of differentiation milieu with only one of them. Findings show that HA in solution with chondroitin may create more intermolecular hydrogen bonds than when in a homogeneous solution, thus increasing HA stability. Indeed, the viscoelastic properties of the synovial fluid can improve if a HA/chondroitin network is present (Andrysiak et al., 2018). In line with these findings, we used HA/BC complex that were obtained with a thermal stabilization procedure that optimizes the formation of hydrogen bonds among polymers (De Rosa et al., 2012). In this context, the contemporary *in vitro* supplementation of both GAGs may represent a more physiological environment for chondrocyte differentiation.

Some scientists suggest that GAGs have a “sulfation code” whereby they encode functional information (Gama et al., 2006; Nandini and Sugahara, 2006; Hussein et al., 2020). For this reason, we used bCS and fCS that have different lengths and sulfation patterns and compared them with BC that is unsulfated. The fCS contains the higher amount of disulfated disaccharide and a 4S/6S ratio of 0.73, while bCS has 4S/6S ratio of 1.61 (Restaino et al., 2019).

We did not observe significant differences in the pro-differentiation capacity of bCS and fCS. In contrast, the BC that has no sulfation showed the best performance when combined with HA. The HA/BC hydrogel induced chondrocyte differentiation with high expression of Type II collagen and reduced expression of Type I collagen and MMP13. Furthermore, the cell-death levels were minimal in this experimental condition. This result is in line with our previous finding showing that BC can preserve the chondrocyte phenotype in cultures of human nasal chondrocytes (Stellavato et al., 2016).

It remains to be determined why BC, which has no sulfation moiety, shows higher differentiation performance than sulfated chondroitin molecules. Indeed, findings show that chondroitin chains with variable sulfation patterns might form distinct domains, which promote interaction with other biological macromolecules, such as growth factors. For this reason, it is even more interesting to find this peculiar activity for BC, that, on the one hand, is behaving similarly to both HA and CS, being different from the former in having acetylated galactosamine in place of glucosamine on the repeating unit, while, on the other hand, not presenting any HSO_3_^–^ group, thus being diversely charged (or less charged).

The current research is one of the first reports about BC (Russo et al., 2020) and hence the in-depth knowledge of its biochemical features, which are the main drivers toward bioactivity, are still under investigation. It can be argued that the unsulfated chondroitin is very similar to low MW, HA, differing in the repetitive unit only (acetylated glucosamine in the HA versus acetylated galactosamine in the chondroitin). In principle, this may suggest a cell-macromolecule crosstalk quite similar to the one between HA and the cell surface. In this context. It should be underlined that the MW is a discriminating factor in HA bioactivity (D’Agostino et al., 2017) and this might be the basis for further studies to unravel a more detailed biochemical cascade for BC. A recent finding provided data about a multicenter, open label, pilot study aiming at treatment of OA patients with an intra-articular injection of a hybrid complex of HA and BC (Papalia et al., 2020). The preliminary results evidenced that treatment was well tolerated and effective in pain relief. Our finding on the effect of combination of HA and BC on chondrocyte differentiation may contribute to dissect molecular mechanisms that promote the success of treatment described in the clinical trial.

## Conclusion

Finally, we can assert that thanks to the presented results, sulfated chondroitins, and above all biotechnological unsulfated ones, are improving MSCs’ commitment when timely used, and their combination with HA is more powerful, eventually showing synergic effects in the GAGs terminal differentiation model. These GAGs may then be used in *in vitro* expansion and differentiation of stem cells before implantation, permitting higher cell viability and lower senescence (as previously obtained) and more extensive differentiation.

## Materials and Methods

### Materials

Extractive chondroitin sulfate was kindly donated by IBSA (Switzerland). and was characterized as previously reported (Restaino et al., 2019). The fCS was 96% pure, on a dry basis, with <2% keratan sulfate, 36 kDa MW, as analyzed at size exclusion chromatography with triple detector (SEC-TDA), and a polydispersity of 1.2. The bCS, extracted from bovine trachea, presented a purity grade higher than 95%, on a dry basis, contained about 2.2% of keratan sulfate, a 19 kDa MW at SEC-TDA and polydispersity of 1.23. The BC was purified from the fermentation broth in our lab, and extensively characterized, it was 97% pure on a dry basis, as revealed by capillary electrophoresis, and has a 35KDa MW with a 1.1 polydispersity index. In addition, the endotoxin content was lower than 0.05 EU/mg. The HA was from Altergon (Italy) and is a highly purified fermentative product (Shyalt) presenting about 1500 KDa with 1.15 polydispersity. The hydrogels were prepared by dissolving 10 mg/mL of each GAG in PBS and then autoclaving them. The combination of HA and CS was obtained by mixing overnight and then autoclaving the gel. The complexes containing HA/BC were obtained, after extensive overnight mixing, following the thermal treatment described before (Stellavato et al., 2016; Restaino et al., 2019).

### MSC Cultures

The experimental procedures followed the rules approved by the Ethics Committee of the Luigi Vanvitelli Campania University (Italy). Patients were informed of the research and gave permission for the use of biological samples. Bone marrow was harvested from three healthy donors. We separated cells using a Ficoll density gradient (GE Healthcare, Milan, Italy), and the mononuclear cell fraction was collected and washed in PBS. We seeded 1 to 2.5 × 10^5^ cells/cm^2^ in alpha-minimum essential medium (alpha-MEM) containing 10% fetal bovine serum (FBS) and 1 ng/ml beta-fibroblast growth factor (β-FGF). After 72 h, non-adherent cells were discarded and adherent cells were further cultivated to confluency. We verified that, under our experimental conditions, the bone marrow stromal cultures contained MSCs that fulfilled the three criteria proposed to define MSCs (Dominici et al., 2006). All experiments were carried out on MSC cultures at passage three when senescence phenomena were minimal (Alessio et al., 2018a).

### *In vitro* Chondrogenic Differentiation and Safranin O Staining

We plated MSCs (5 × 10^3^ cell/cm^2^) in chondrogenic medium (DMEM, 1% FBS, 0.1 mM dexamethasone, 50 nmol/L ascorbate-2-phosphate, 10 ng/mL human TGF-β1, ITS 1X) (Solchaga et al., 2011). We changed the medium every 3 days. The differentiation procedure lasted 28 days and we designated this as the reference method (see results). Safranin O staining was used to identify glycosaminoglycan formation on the cell surfaces. Briefly, we fixed cells in acetone/methanol solution (at 4°C) for 3 min and then we incubated cells at room temperature (RT) in Safranin O solution (0.1%) for 5/10 min. Stained cultures were analyzed under the light microscope. All reagents were from Sigma-Aldrich (St. Louis, MO, United States).

### Immunohistochemistry

The cell pellets were collected by centrifugation at 2,000 rpm for 5 min at RT and resuspended in 4% formaldehyde (Sigma-Aldrich) solution for 15 min at RT. The pellets were washed three times with PBS 1X and resuspended in permeabilization buffer 0.3% Triton X-100 (Roche Diagnostics, Mannheim, Germany) for 10 min in ice. Pellets were then collected by centrifugation and resuspended in blocking buffer (5% FBS and 0.1% Triton X-100 in PBS) for 1 h at RT. Subsequently, the antibodies COL1 (Cod. E-AB-63704 Elabscience, Houston, TX, United States), COL2 (cod. E-AB-60340 Elabscience), or MMP13 (Cod. E-AB-60365 Elabscience) were utilized, according to the manufacturer’s protocols. The FITC-conjugated secondary antibody was obtained from ImmunoReagents (Raleigh, NC, United States). Nuclear staining was performed using DAPI mounting medium (ab104139, Abcam, Cambridge, United Kingdom) and microscopy images were captured under a fluorescence microscope (DM2000, Leica, Wetzlar, Germany). The mean pixel intensity for antibodies was quantified using Quantity One 1-D analysis software (Bio-Rad, Hercules, CA, United States).

### RNA Extraction and RT-qPCR

Expression levels of aggrecan (AGN), Types I and II collagen (COL1A2 and COL2A1), SRY-box transcription factor 9 (SOX9) and hyaluronan synthase 1 (HAS1) were assayed through quantitative real-time PCR (qRT-PCR). A full description of the gene expression method has already been reported (Stellavato et al., 2016). Briefly, total RNA was isolated from cells using TRIzol (Invitrogen, Italy). 1 μg of DNase-digested total RNA (DNA-free kit; Ambion-Applied Biosystems, CA, United States) was reverse-transcribed to cDNA using the Reverse Transcription System Kit (Promega, Italy). The iQ SYBR B Green Supermix was used (Bio-Rad, Italy) to amplify specific genes using appropriate primer pairs. All reactions were performed in triplicate, and the relative expression of specific mRNA was normalized to glyceraldehyde-3-phosphate dehydrogenase (GAPDH) housekeeping gene. The fold-change was calculated using the comparative threshold method (DDCt = difference in DCt between GAG-treated cells and control) and the results are expressed as the normalized fold expression using the quantification of 2^–ΔΔCt^ method.

### Statistical Analysis

Statistical significance was evaluated using ANOVA analysis followed by Student’s *t*-test and Bonferroni’s test. A mixed-model variance analysis was used for data with continuous outcomes. All data were analyzed with a GraphPad Prism version 5.01 statistical software package (GraphPad, La Jolla, CA, United States).

## Data Availability Statement

The raw data supporting the conclusions of this article will be made available by the authors, without undue reservation.

## Ethics Statement

The studies involving human participants were reviewed and approved by School of Medicine – Campania University. The experimental procedures were performed according to the rules approved by the Ethics Committee of the Luigi Vanvitelli Campania University (887/2008).

## Author Contributions

UG and CS conceptualization, supervision, and writing—review and editing. NA, GD, AS, DC, and VV data curation. CS funding acquisition. AS, VV, DA, and NA investigation. AS, DA, DC, and NA methodology. NA, DC, DA, and GD validation. NA writing—original draft. All authors contributed to the article and approved the submitted version.

## Conflict of Interest

The authors declare that the research was conducted in the absence of any commercial or financial relationships that could be construed as a potential conflict of interest.

## Supplementary Material

Supplementary File 1 Chondrocyte differentiation markers in MSC cultures treated with reference differentiation method (DM). The picture shows the mRNA levels of aggrecan (AGN), Type I collagen (Col I), Type II collagen (Col II), and SOX9 in MSCs induced to chondrocyte differentiation with DM for 28 days. Data are normalized to mRNA levels observed in undifferentiated cultures. For each mRNA, the expression level observed in undifferentiated cultures is set at 1, its decrease or increase in the DM conditions is expressed as fold change. Data are reported with standard deviation (*n* = 3). The symbol ^∗^ indicates the statistical difference between the differentiated and the untreated cultures (^∗∗^*p* < 0.01).

## Abbreviations

ACI: autologous chondrocyte implantation
BC: bio-fermentative unsulphated chondroitin
CS: chondroitin sulfate
DM: differentiating medium
FBS: fetal bovine serum
GAG: glycosaminoglycan
HA: hyaluronic acid
IHC: immunohistochemistry
MSC: mesenchymal stromal cells
MW: molecular weight
OA: osteoarthritis
PBS: phosphate buffer saline solution
qRT-PCR: quantitative real-time PCR
SEC-TDA: size exclusion chromatography with triple detector.
