# Supplementary figures and images for: Timely Supplementation of Hydrogels Containing Sulfated or Unsulfated Chondroitin and Hyaluronic Acid Affects Mesenchymal Stromal Cells Commitment Toward Chondrogenic Differentiation
Source: Front Cell Dev Biol. 2021 Apr 12;9:641529. doi: 10.3389/fcell.2021.641529 (PMC8072340; doi:10.3389/fcell.2021.641529)

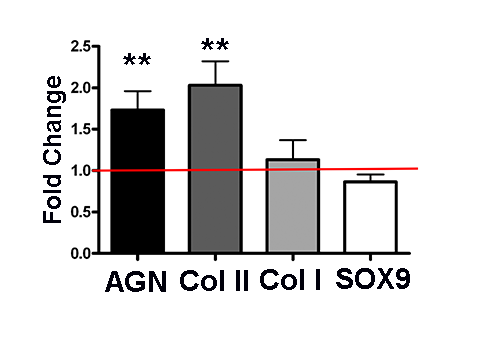

Supplement: Supplementary File 1 — Chondrocyte differentiation markers in MSC cultures treated with reference differentiation method (DM). The picture shows the mRNA levels of aggrecan (AGN), Type I collagen (Col I), Type II collagen (Col II), and SOX9 in MSCs induced to chondrocyte differentiation with DM for 28 days. Data are normalized to mRNA levels observed in undifferentiated cultures. For each mRNA, the expression level observed in undifferentiated cultures is set at 1, its decrease or increase in the DM conditions is expressed as fold change. Data are reported with standard deviation (n = 3). The symbol ∗ indicates the statistical difference between the differentiated and the untreated cultures (∗∗p < 0.01). [file Image_1.tif]
